# Staged surgical treatment for severe and rigid scoliosis

**DOI:** 10.1186/1749-799X-3-26

**Published:** 2008-07-09

**Authors:** Shi Yamin, Li Li, Wei Xing, Gao Tianjun, Zhang Yupeng

**Affiliations:** 1 Department of Orthopedics, The 1st Affiliated Hospital to the General Hospital of PLA, Beijing, PR China

## Abstract

**Background:**

A retrospective study of staged surgery for severe rigid scoliosis. The purpose of this study was to evaluate the result of staged surgery in treatment of severe rigid scoliosis and to discuss the indications.

**Methods:**

From 1998 to 2006, 21 cases of severe rigid scoliosis with coronal Cobb angle more than 80° were treated by staged surgeries including anterior release and halo-pelvic traction as first stage surgery and posterior instrumentation and spinal fusion as second stage. Pedicle subtraction osteotomy(PSO) was added in second stage according to spine rigidity. Among the 21 patients, 8 were male and 13 female with an average age of 15.3 years (rang from 4 to 23 years). The mean pre-operative Cobb angle was 110.5° (80°-145°) with a mean spine flexibility of 13%. Radiological parameters at different operative time points were analyzed (mean time of follow-up: 51 months).

**Results:**

External appearance of all patients improved significantly. The average correction rate was 65.2% (ranging from 39.8% to 79.5%) with mean correction loss of 2.23° at the end of follow-up. No decompensation of trunk has been found. Mean distance between the midline of C7 and midsacral line was 1.19 cm ± 0.51. Two patients had neurological complications: one patient had motor deficit and recovered incompletely.

**Conclusion:**

Staged operation and halo-pelvic traction offer a safe and effective way in treatment of severe rigid scoliosis. Patients whose Cobb angle was more than 80° and the flexibility of the spine was less than 20% should be treated in this way, and those whose flexibility of the spine was less than 10% and the Cobb angle remained more than 70° after 1st stage anterior release and halo-pelvic traction should undergo pedicle subtraction osteotomy (PSO) in the second surgery.

## Background

Excellent outcomes of hemi vertebra excision, vertebral body resection, and spinal osteotomy have been reported for angular kyphosis or kyphoscoliosis. However, their safety and effectiveness of these procedures have not been estimated. It would be difficult to correct severe and rigid spinal deformities satisfactorily by a single procedure in consideration of the neurological safety. In consequence, staged surgeries have been widely used in the treatment of severe rigid scoliosis. Nevertheless, in few papers the method of anterior releases followed by halo-pelvic traction has been mentioned.

There is a high risk in the surgical correction for severe rigid scoliosis. A 5.3% incidence of permanent neurological injury has been reported by Dutoit and the incidence of transient neurological deficit was as high as 46% in Luque's records. [1,2] Staged surgery has been used in the treatment of severe rigid scoliosis to prevent neurological compromise. The conventional staged surgery consists of anterior release as first stage procedure and posterior spinal fusion and instrumentation as second stage [3-6]. Nevertheless, in few papers the method of anterior releases followed by halo-pelvic traction has been mentioned, and the indication of staged surgical methods is discrepancy. With the development of the surgical and anesthesia technology, combined anterior and posterior procedure has been used in recent decade. However, its advantage of reduced hospital stay and costs was not comparable to its higher complication rate. [7,8] This paper evaluated the outcome of 21 cases with severe and rigid scoliosis retrospectively treated with staged surgery and the indication was discussed.

## Materials and methods

From 1998 to 2006, 21 cases of severe rigid scoliosis were treated with staged surgery. among the 21 patients, 8 were male and 13 female with an average age of 15.3 years (rang from 4 to 23 years). The scoliosis was classified as congenital in 11 cases, idiopathic in 7 and neuroinomatosis in 2. The mean preoperative coronary Cobb angle was 110.5° (range from 80° to 145°), the mean Cobb angle was 94.5° (70°-133°) on suspension view. Flexibility was used to estimate the rigidity of the curve, it means (Preoperative Cobb's angle – Bending Cobb's angle)/Preoperative Cobb angle × 100%. The curve was considered stiffness when it was more than 30%. The mean flexibility of the spine was 13% (range 1.5% to 27.3%)in this group. 2 cases of congenital scoliosis were confirmed diastematomyelia in the spinal canal by CT and MRI. Mild or severe limited dysfunctions of ventilation existed in all the cases.

All cases were grouped into two. 13 cases in group A were performed with anterior release and halo-pelvic traction in first stage; and then posterior spinal fusion and instrumentation in second stage. In group B, 9 cases were treated with the same procedure as in group one in the first stage; and then posterior spinal fusion and instrumentation plus wedge resection. The vertebral osteotomy was done from T7 to L2. SEP and wake-up test were used in all patients during operation.

### Principal Surgical Techniques and Highlights

#### Anterior spine release and deformity correction with halo-pelvic distraction apparatus

Apex and adjacent vertebra were exposed from convex side through thoracic pathway, usually only 4 to 6 discectomy could be performed because of the limitation of exposure. Significant abnormal intervertebral mobility should be confirmed. during operation.

4 pelvic screws were inserted at sites 2 to 2.5 cm posterior-inferior to bilateral anterior-superior iliac spine and posterior superior iliac spine respectively, which were linked to pelvic ring and fixed. 4 cranial screws were inserted at sites 1 cm superior-lateral to bilateral arcus superciliaris and 2 cm superior to bilateral mamillary process respectively.4 connector bars were linked between halo and pelvic ring.

Lengthening of Halo-pelvic distraction apparatus began 3 to 5 days after operation, with the extent of 2 times a day and 3 to 5 mm each time. The indications of traction limit include early appearance of clinical symptoms of cranial nerves or spinal cord, muscular pain, gastrointestinal symptoms which affected food intake even if the lengthening was stopped, and severe pin tract infection caused by the loose of screws.

#### Insertion of segmental pedicle screw system and correction of deformity

During the second stage pedicle screws were inserted continuously or interruptedly in the concave side of the stability region [9], 2 screws should be inserted consecutively at up end and low end and apex vertebra to decrease the regional stress and strengthen correction force on the apex vertebra. Fasten screws were tapped into the tugs of pedicle screws after the pre-bending rod was put into tugs of pedicle screws. Correction force to the spine was achieved by rotating of the rod. The direction of rod rotation was based on the types of scoliosis. The rod should be rotated from the convex side to concave side in thoracic scoliosis to transform the scoliosis in coronal plan to kyphosis in sagital plan. For the lumbar scoliosis, the rod should be rotated from concave side to convex side to transform the scoliosis in coronal plan to lordosis in sagital plan. The rod should be rotated in the same direction in thoracolumbar double-curve scoliosis to achieve correction of double-curve scoliosis deformity and restoration of sagital curve of thoracic and lumbar spine.

#### Wedge-shape osteotomy of apex vertebra and deformity correction

Single vertebra or intervertebral disk space can be selected as the center of wedge-shape osteotomy according to the type of apex vertebra. To prevent possible neurological symptoms caused by shrinkage of spinal cord, adjacent half-laminectomy superior and inferior to the osteotomy site should be performed for canal decompression. Abnormities in spinal canal (bony crista or septation) or spinal stenosis should be managed before osteotomy and deformity correction. Exposure of osteotomy region began from the convex side of apex vertebra, with the soft tissue dissected sub-periosteally. The anterior-lateral side of the apex vertebra or intervertebral disk space should be exposed completely. According to the preoperative design, osteotomy of apex vertebra or adjacent vertebras with apex center in disk space was performed.

The nerve root at the level of excision should be identified and protected, particularly in the lumbar region, but some severe cases in the thoracic region, the nerve root had to be cut and left in place, this helps protect the dural tube because the curette can be levered safely on this bone and avoiding traction forces to the cord. And then, osteotomy site was temporally fixed with rod to prevent abnormal movement. The osteotomy of concavity side was performed in the same way. Temporary rod in the convex side was removed after the pre-bending rod was put in concave side. The osteotomy space in the convex side was gradually closed by slowly rotating the rod in the concave side, then pre-bending rod in the convex side was fixed, the osteotomy space in the convex side might be closed by proper compression the adjacent pedicle screws on the basis of the size of the osteotomy space and the extent of spinal cord shrinkage. Some epidural and bone bleeding is to be expected and can be controlled with gel foam, bone wax, and bipolar cautery.

## Results

In a total of 21 patients, the average Cobb angle was 62° (range 40° to 89°) after first-stage release and traction surgical procedures, the average correction rate was 44.2%(range 23.9 to 63.9%). After second-stage correction with instrumentation, the average Cobb angle was 39.4° (range 22° to 73°). The average correction rate was 65.2% (range 39.8% to 79.5%).

Preoperative deformity degree and clinical effects were investigated and analyzed with SPSS 11.0(SPSS, Inc., Chicago, IL) (Tab 1): Because of heterogeneity of variance in age of two groups, WilCoxon rank sum test was used and demonstrated no significant difference in age (P > 0.05), analysis of variance demonstrated no significant difference in preoperative Cobb angle (P > 0.05), curve correction rate after traction surgical procedure (P > 0.05), But there was significant difference in spine flexibility (P < 0.05) between the 2 groups. (Table 1)

**Table 1:** Evaluation of the outcome in staged surgical methods for severe rigid scoliosis

Group	Cases	Age	Pre-OP Cobb	Suspension Cobb	Flexibility^▲^	PO-Traction Cobb	Traction Rate	PO-OP Cobb	Correction rate(%)
Staged Operation	12	4~23 (14.4)	80~145° (112.5°)*	70~133° (94.1°)	1.5~27.3% (14.9%)	40~89° (60.0°)	23.9~63.9% (47.8%)	22~58° (40.6°)	50.4~79.5% (65.4%)
Staged+ Osteotomy	9	6~21 (16.8)	90~132° (107.5°)	78~124° (95.5°)	5.6~16.1% (10.1%)	46~85° (65.0°)	27.8~54.9% (38.9%)	30~49° (37.5°)	59.3~72.3 (65.0%)

Total	21	4~23 (15.3)	80~145° (110.5°)	70~133° (94.5°)	1.5~27.3% (13.0%)	40~89° (62.0°)	23.9~63.9% (44.2%)	22~73° (39.4°)	39.8~79.5% (65.2%)

*Significant difference (p < 0.05) between two groups in the flexibility of the spine and no significant difference in age, Cobb angle and correction rate.

Number in sign of aggregation is average value.

▲ Flexibility degree = (Pre-OP Cobb- Hang-up Cobb)/Pre-OP Cobb*100%

A total of 21 patients were followed up after operation. The average follow up period was 51 months (range 5 to 81). At one year after surgery, 20 patients' showed a solid segments fusion with no hardware failure. Average loss of correction rate was 2.1% (range 1.3% to 6.1%). No decompensation findings have been observed. Mean distance between the midline of C7 and midsacral line was 1.16 cm ± 0.54. One pedicle screw come out at 3 year after surgery, the pseudoarthroses were resected in the revision surgery. The rate of neurological complications was 9.5% (2/21 patients); and these two patients all were subjects of congenital scoliosis. One patient showed temporary paraplegia at the level of the osteotomy site, but completely recovered within 10 days after the additional decompression of vertebral canal and treated with hormone and dehydration; the other one showed permanent neurological deficit. At both lower extremities during the derotation procedure. He recovered to III-IV muscle grade, but there were not significant changes at 67 months after surgery.

Radiographic assessment for sagittal balance was performed by measuring thoracic kyphosis, lumbar lordosis, distance between the vertical line on anterosuperior point of T1 and that of S1, and sacral inclination. Clinical outcomes were assessed by questionnaire measuring changes in physical function, indoor activity, outdoor activity, psychosocial activity, pain, and patient satisfaction with surgery.

The mean trunk shift in global sagittal balance was 21 mm before surgery, becoming 3 mm after surgery.

Final follow-up radiograph showed an increase in lumbar lordosis from 20.1 degrees to 44.6 degrees (an increase of 24 degrees), whereas thoracic kyphosis remained stable from 87 degrees to 54 degrees. Sagittal imbalance significantly improved, whereas sacral inclination increased from 8 degrees to 24 degrees. Satisfactory clinical outcome was achieved; however, clinical improvements did not correlate with changes in radiological measurements.

## Discussion

The therapeutic efficacy of scoliosis is influenced by many factors, such as the severity of deformity, spine flexibility, patient's age, type of deformity, and combined other deformities. Severe scoliosis is more difficult to treat than usual ones. As the spine deformity is severe and stiff, and the spinal cord has poor tolerance to the traction. It is difficult to complete the correction, and the probability of nerve deficit increases. Moreover, because severe scoliosis is usually combined with heart or lung disfuncitons, the operation is of relatively high risk.

**Figure 1:**
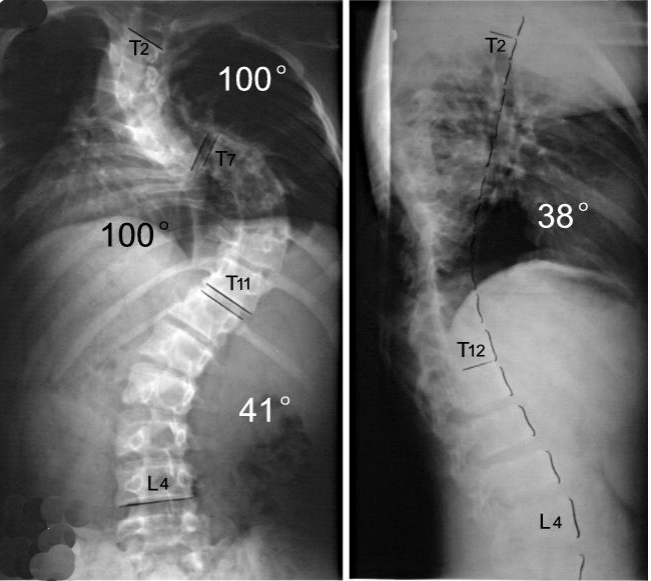
M,12Y, neurofibromatosis scoliosis, double thoracic curve.

**Figure 2:**
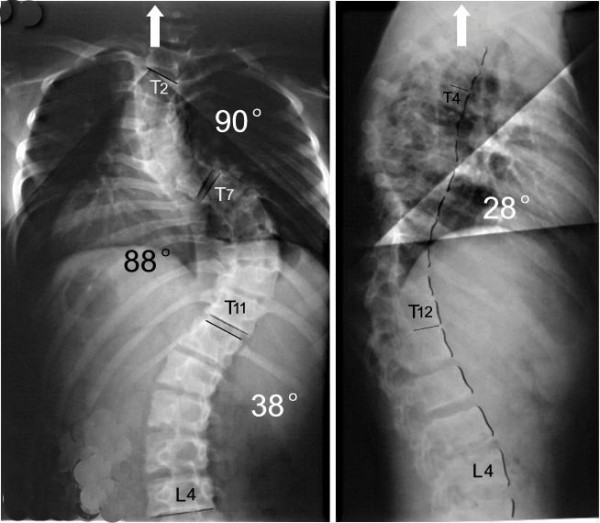
Suspension view shows the flexibility of spine.

**Figure 3:**
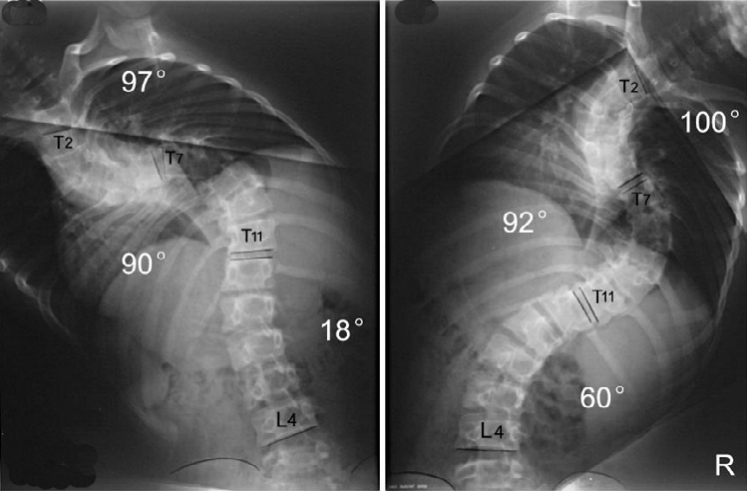
Bending view shows the change of deformity.

**Figure 4:**
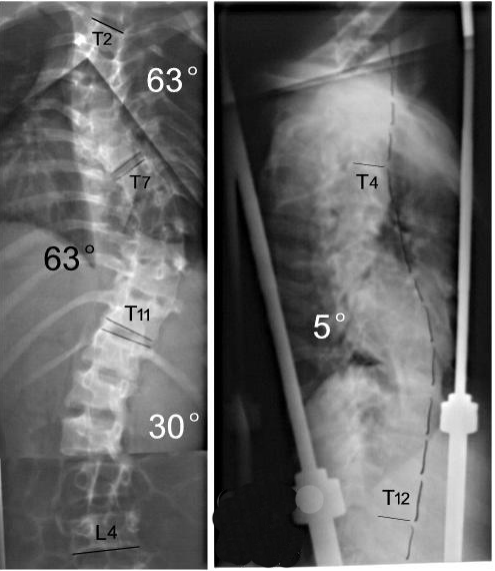
18 days after anterior release and halo-pelvic traction. The correction rate is 37%.

**Figure 5:**
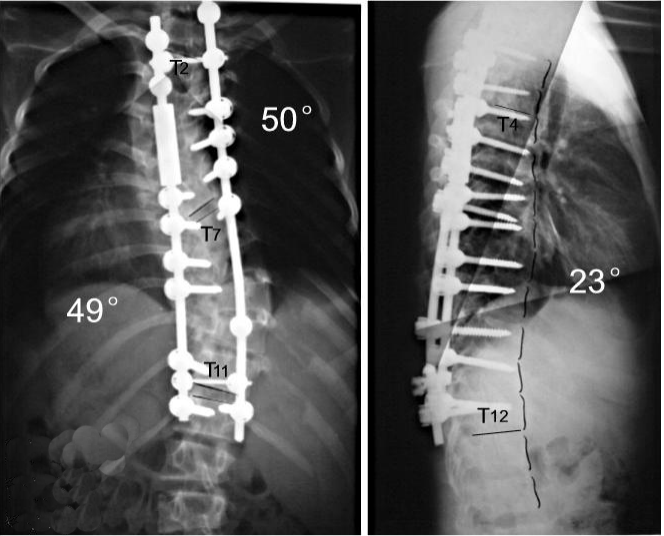
The correction rate is 51% after second operation.

**Figure 6:**
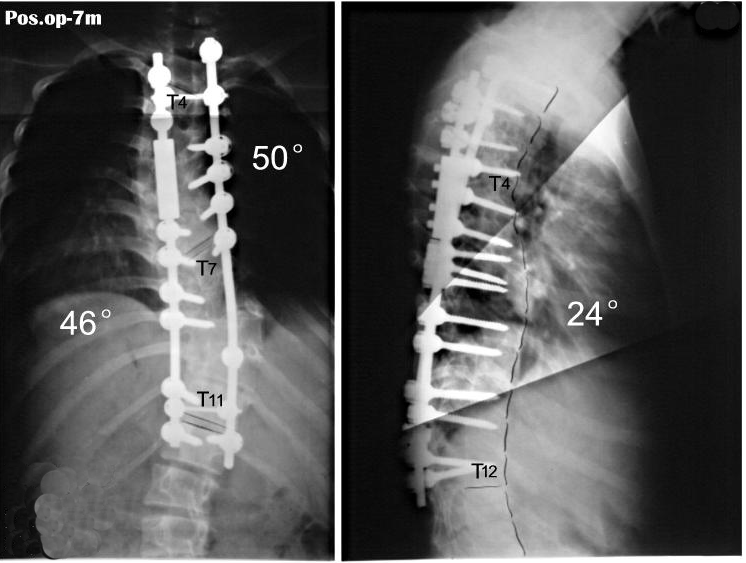
No correction loss at follow-up 6 months later.

**Figure 7:**
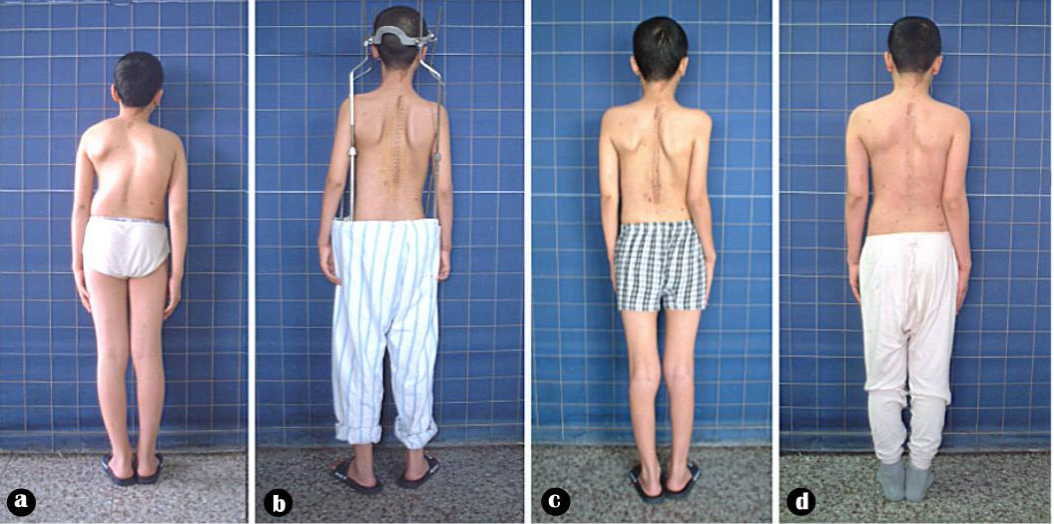
Body image a: pre-operation b: after anterior release and halo-pelvic traction. c: after second stage correction d: follow-up 6 months later.

**Figure 8:**
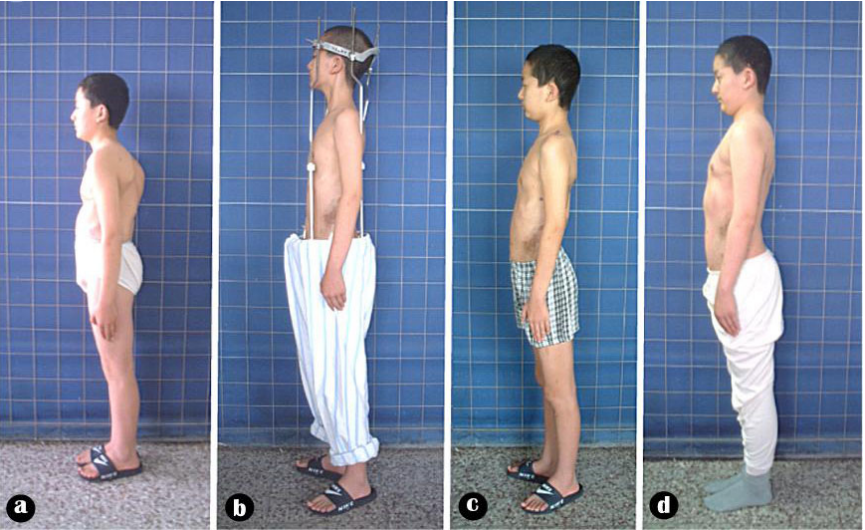
Body image a: pre-operation b: after anterior release and halo-pelvic traction. c: after second stage correction d: follow-up 6 months later.

The scoliosis severity is the chief factor that may affect the outcomes of deformity correction. Usually, if the coronal Cobb's angle is more than 80° and the spine flexibility is less than 20%, anterior loosen combined with halo-pelvic traction should be accepted, then followed by posterior correction in the second stage. (Fig 1, 2, 3, 4, 5, 6, 7, 8) For the patients with neurological symptoms preoperatively, halo-pelvic traction can also be used to prevent the neurological deficit from aggravating. As the spinal cord can creep slowly, and the halo-pelvic traction can provide gentle correction on spine, a good correction can be achieved. Furthermore, even if neurological complication appears during traction, the halo-pelvic device can be adjusted to relieve it. Therefore, although the halo-pelvic device has some disadvantages (e.g. hardware complicated and nursing problems), it is an alternative method to the prevention of neurovascular complications in the treatment of severe and rigid scoliosis without any major or permanent neurological deficit.

**Figure 9:**
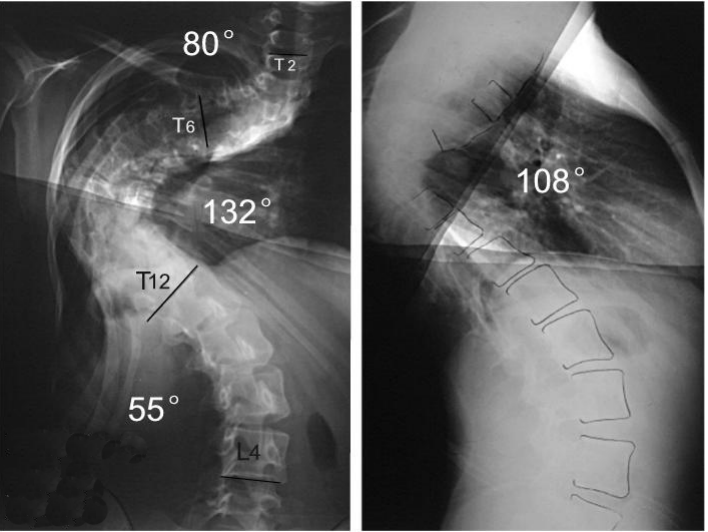
M, 21Y, Idiopathic kyphoscoliosis.

**Figure 10:**
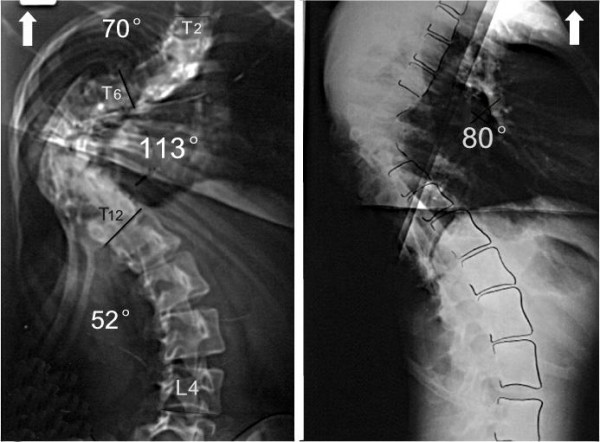
Suspension view shows the flexibility of spine.

**Figure 11:**
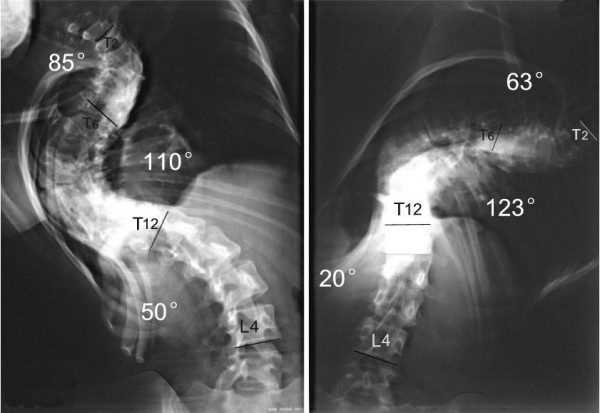
Bending view shows the change of deformity.

**Figure 12:**
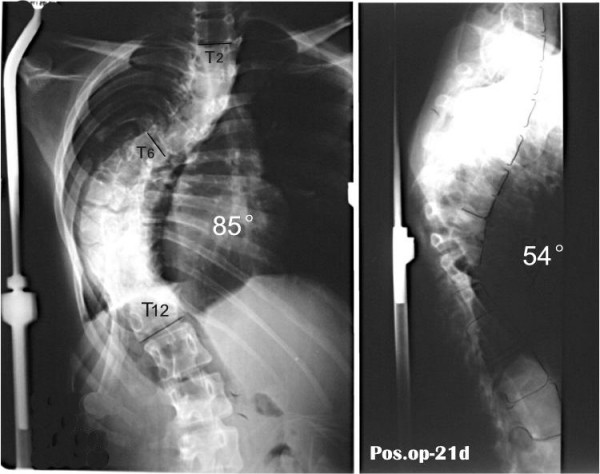
days after anterior release and halo-pelvic traction. The correction rate is 35.6% and 50%.

**Figure 13:**
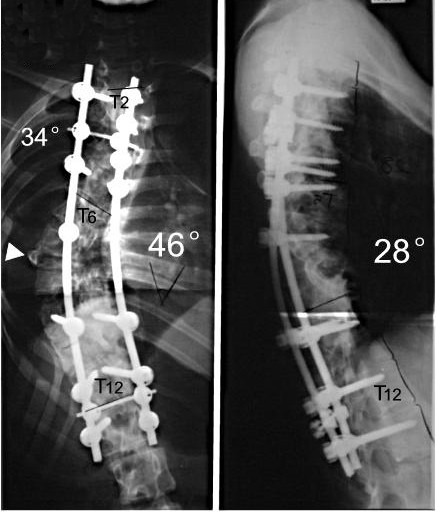
The correction rates are 65.2% and 74.1% after second stage osteotomy and instrumentation.

**Figure 14:**
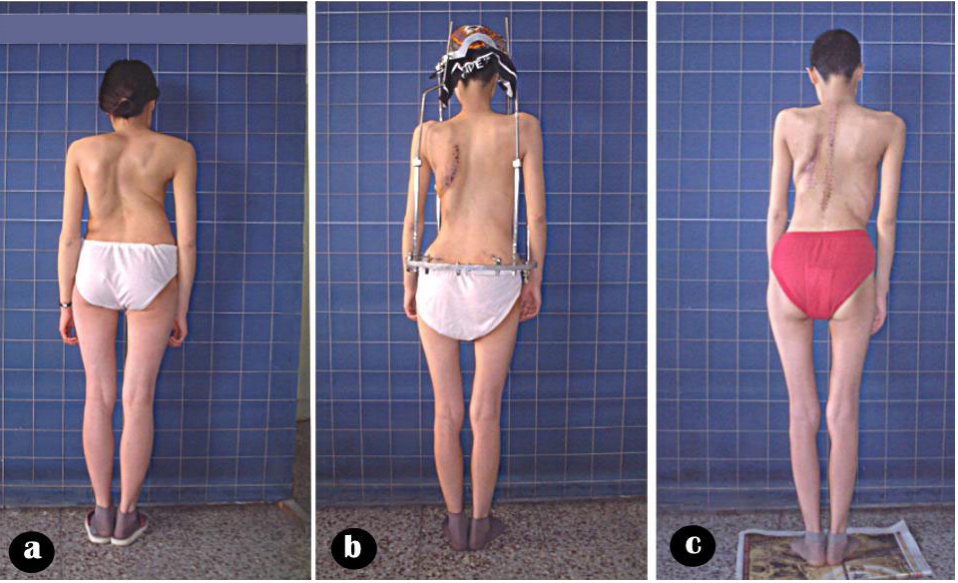
Body image a: pre-operation b: after anterior release and halo-pelvic traction. c: after second stage correction.

**Figure 15:**
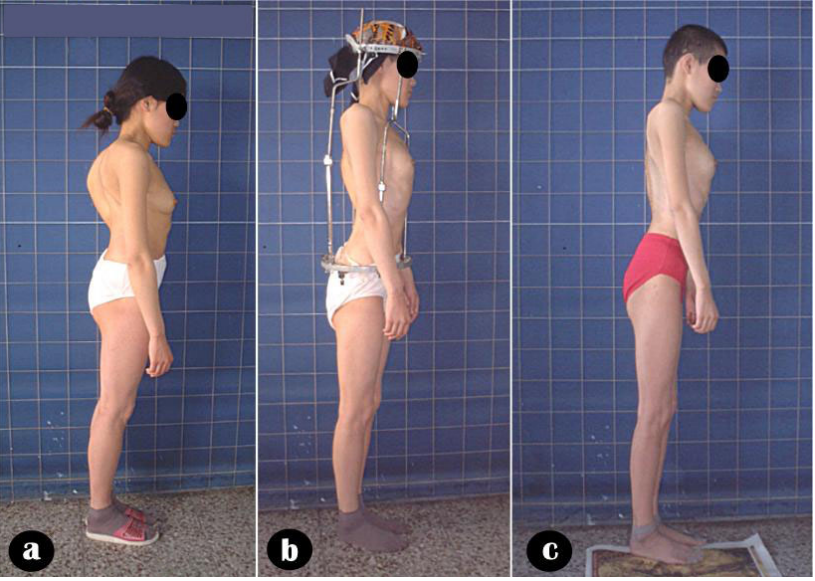
Body image a: pre-operation b: after anterior release and halo-pelvic traction. c: after second stage correction.

For those cases with the spine flexibility less than 10%, remained Cobb angle more than 60~70° after halo-pelvic traction, nerve deficit reappear in the later stage of traction, and most severe congenital scoliosis up the adolescent age, it is difficult to get a good correction only using the posterior bar rotation in the second stage, so osteotomy should be used(Fig 9, 10, 11, 12, 13, 14, 15). According to this research, the spine flexibility of the first group is obviously less than the second group; however, the correction rate has no significant difference between the two teams. It is proved that osteotomy is very effective for the correction of the severe scoliosis.

Though osteotomy is useful in the treatment of scoliosis, it can bring some complications, especially the nerve deficit. Bradford etc. had performed 24 cases of osteotomy, and 3 of those had nerve deficit (12.5%) [10]. Among the 3 cases, muscle strength of ankle flexion weakened in 2 cases, and quadriceps femoris weakened in 1 case. Considering the possible rather too big local lumbar curve, vertebral canal decompression was performed, and a good recovery was achieved 6 months later. Berven etc. reported a series of 13 cases undergoing osteotomy [11]. Leg palsy happened in 4 cases (30.8%). These cases got complete reablement half a year later. As to our research, of the 2 cases with leg sensory motor dysfunction, 1 case had undergone osteotomy. The reason was probably that too big range of osteotomy, the spinal cord shrinked after the gap closed, so the vertebral canal was relatively narrow. For this case, SEP showed the latent period increased (>30%), and the wave amplitude decreased (>50%) during operation monitoring. The symptoms disappeared 1 week later after the enlarged decompression of vertebral canal and other treatment postoperatively. All of the cases in this research and other literatures had no nonreversible nerve deficit due to osteotomy.

Current literatures say on the standard of care for severe scoliosis that the treatment approach is different to the subjects in this paper. Dr.Luhmann SJ, and Dr.Lenke LG recently address that anterior and posterior spinal fusion of large thoracic curves allows greater coronal correction of thoracic curves between 70 degrees and 100 degrees, when compared with PSF alone using thoracic hook constructs, but not with the use of thoracic pedicle screw constructs[12]. Scoliosis surgeons not using pedicle screw constructs need to decide if the modest improvement in coronal correction with a combined approach justifies its routine use in this patient population.

Dobbs MB and Lenke LG said in their patient population with often restrictive preoperative pulmonary function [13], a posterior-only approach with the use of an all-pedicle screw construct has the advantage of providing the same correction as an anterior/posterior spinal fusion, without the need for entering the thorax and more negatively impacting pulmonary function.

One of the main technical problems we encountered in this mode of treatment is how to protect spinal cord during pedical subtraction osteotomy. Procedures such as osteotomy may be associated with a significant threat of neurological complications. In my experiences, we must stick to 3 key points (1) A temporary rod must be used inserting to the convex side after osteotomy on this side to prevent shear forces; (2) Do the additional decompression after derotation and closing of the osteotomy gap to confirm there is no compression to the cord; (3) SEP or MEP monitoring and wake-up test during and after the derotation correction. Because experiences with this procedure are fairly recent, longer follow-up is required to confirm whether this technique is reliable and efficacious.

## Conclusion

As the spinal cord of the cases with severe rigid scoliosis has poor tolerance to the traction, there is a high risk during the correction, and the staged operation, especially the Halo-pelvic distraction is an effective method to prevent neurological complications. Usually, if the coronal Cobb's is more than 80°, and the flexibility is less than 20%, anterior release with halo-pelvic traction should be suggested, and followed by posterior correction with instrumentations in the second stage. For the severe and rigid cases with the flexibility less than 10%, and the magnitude of curve more than 60~70° after halo-pelvic traction, the patients should undergo pedical subtraction osteotomy(PSO) with instrumentations in the second surgery.

## Consent

Written informed consent was obtained from the patient for publication of this case report and accompanying images.

## Authors' contributions

SY in charge of all the study and perform all operations, LL perform all operations and complete the manuscript, WX, GT, ZY complete data collection and radiograph measurement.
